# An inherited duplication at the gene *p21 Protein-Activated Kinase 7* (*PAK7*) is a risk factor for psychosis

**DOI:** 10.1093/hmg/ddu025

**Published:** 2014-01-28

**Authors:** Derek W. Morris, Richard D. Pearson, Paul Cormican, Elaine M. Kenny, Colm T. O'Dushlaine, Louis-Philippe Lemieux Perreault, Eleni Giannoulatou, Daniela Tropea, Brion S. Maher, Brandon Wormley, Eric Kelleher, Ciara Fahey, Ines Molinos, Stefania Bellini, Matti Pirinen, Amy Strange, Colin Freeman, Dawn L. Thiselton, Rachel L. Elves, Regina Regan, Sean Ennis, Timothy G. Dinan, Colm McDonald, Kieran C. Murphy, Eadbhard O'Callaghan, John L. Waddington, Dermot Walsh, Michael O'Donovan, Detelina Grozeva, Nick Craddock, Jennifer Stone, Ed Scolnick, Shaun Purcell, Pamela Sklar, Bradley Coe, Evan E. Eichler, Roel Ophoff, Jacobine Buizer, Jin Szatkiewicz, Christina Hultman, Patrick Sullivan, Hugh Gurling, Andrew Mcquillin, David St Clair, Elliott Rees, George Kirov, James Walters, Douglas Blackwood, Mandy Johnstone, Gary Donohoe, International Schizophrenia Consortium, SGENE+ Consortium, Francis A. O'Neill, Wellcome Trust Case Control Consortium 2, Kenneth S. Kendler, Michael Gill, Brien P. Riley, Chris C. A. Spencer, Aiden Corvin

**Affiliations:** 1 Department of Psychiatry and Neuropsychiatric Genetics Research Group, Institute of Molecular Medicine, Trinity College Dublin, Dublin 2, Ireland; 2 Wellcome Trust Centre for Human Genetics, University of Oxford, Roosevelt Drive, Oxford OX3 7BN, UK; 3 Broad Institute and Center for Human Genetics Research of Massachusetts General Hospital, Boston, MA 02142, USA; 4 Montreal Heart Institute, Université de Montréal, Montréal, Québec H1T 1C8, Canada; 5 Departments of Psychiatry and Human Genetics, Virginia Institute of Psychiatric and Behavioral Genetics, Virginia Commonwealth University, Richmond, VA 23284, USA; 6 School of Medicine and Medical Science, University College Dublin, Ireland; 7 Department of Psychiatry, University College Cork, Cork, Ireland; 8 Department of Psychiatry, National University of Ireland, Galway, University Road, Galway, Ireland; 9 Department of Psychiatry, RCSI Education and Research Centre, Beaumont Hospital, Dublin 9, Ireland; 10 DETECT Early Intervention in Psychosis Services, Dun Laoghaire, Co. Dublin, Ireland; 11 Molecular and Cellular Therapeutics, Royal College of Surgeons in Ireland, Dublin 2, Ireland; 12 Health Research Board, 73 Lower Baggot St, Dublin 2, Ireland; 13 MRC Centre for Neuropsychiatric Genetics and Genomics, and Neuroscience and Mental Health Research Institute, Cardiff University, Heath Park, Cardiff CF4 4XN, UK; 14 The Mount Sinai Hospital, New York, NY 10029, USA; 15 University of Washington School of Medicine, Howard Hughes Medical Institute, Seattle, WA 98195, USA; 16 Department of Human Genetics, UCLA School of Medicine, Los Angeles, CA 90095, USA; 17 Rudolf Magnus Institute, University of Utrecht, 3584 CG Utrecht, Netherlands; 18 University of North Carolina, Chapel Hill, NC 27599-7264, USA; 19 Department of Medical Epidemiology and Biostatistics, Karolinska Institutet, SE-171 77 Stockholm, Sweden; 20 Molecular Psychiatry Laboratory, Mental Health Sciences Unit, University College London, London WC1E 6BT, UK; 21 Institute of Medical Sciences, University of Aberdeen, Foresterhill, Aberdeen AB25 2ZD, UK; 22 Division of Psychiatry, University of Edinburgh, Royal Edinburgh Hospital, Edinburgh EH10 5HF, UK and; 23 Department of Psychiatry, Queen's University, Belfast BT7 1NN, Northern Ireland

## Abstract

Identifying rare, highly penetrant risk mutations may be an important step in dissecting the molecular etiology of schizophrenia. We conducted a gene-based analysis of large (>100 kb), rare copy-number variants (CNVs) in the Wellcome Trust Case Control Consortium 2 (WTCCC2) schizophrenia sample of 1564 cases and 1748 controls all from Ireland, and further extended the analysis to include an additional 5196 UK controls. We found association with duplications at chr20p12.2 (*P* = 0.007) and evidence of replication in large independent European schizophrenia (*P* = 0.052) and UK bipolar disorder case-control cohorts (*P* = 0.047). A combined analysis of Irish/UK subjects including additional psychosis cases (schizophrenia and bipolar disorder) identified 22 carriers in 11 707 cases and 10 carriers in 21 204 controls [meta-analysis Cochran–Mantel–Haenszel *P*-value = 2 × 10^−4^; odds ratio (OR) = 11.3, 95% CI = 3.7, ∞]. Nineteen of the 22 cases and 8 of the 10 controls carried duplications starting at 9.68 Mb with similar breakpoints across samples. By haplotype analysis and sequencing, we identified a tandem ∼149 kb duplication overlapping the gene p21 *Protein-Activated Kinase 7* (*PAK7*, also called *PAK5*) which was in linkage disequilibrium with local haplotypes (*P* = 2.5 × 10^−21^), indicative of a single ancestral duplication event. We confirmed the breakpoints in 8/8 carriers tested and found co-segregation of the duplication with illness in two additional family members of one of the affected probands. We demonstrate that *PAK7* is developmentally co-expressed with another known psychosis risk gene (*DISC1*) suggesting a potential molecular mechanism involving aberrant synapse development and plasticity.

## INTRODUCTION

Schizophrenia [MIM 181500] is a poorly understood, but severe, heritable mental disorder with a lifetime risk of ∼1%. The emerging genetic architecture includes a spectrum of risk variation from rare mutations of large effect, to common risk variants of small effect [odds ratio (OR) <1.15] which collectively account for at least 25% of susceptibility (1–3). Through genome-wide association study (GWAS) and subsequent meta-analysis, more than 20 independent common loci have been confirmed, but there are likely to be many more (4–6). A much smaller number of rare mutations of moderate or large effect have been identified, but these will be particularly important in facilitating dissection of the risk phenotype in model systems (7).

The list of rare, highly penetrant schizophrenia mutations (OR = 2–30) includes recurrent *de novo* copy-number variants (CNVs) involving deletions or duplications of large, genomic regions (>100 kb) (8,9), but also the accumulation of different CNV events at specific loci implicating single genes (*NRXN1* [MIM 600565] (10–12) and *VIPR2* [MIM 601970]) (13,14). Almost all of the confirmed CNVs are also risk factors for other psychiatric or developmental phenotypes [e.g. intellectual disability, attention deficit hyperactivity disorder (ADHD), autism] (15,16). This is in keeping with epidemiological and GWAS data supporting shared genetic liability between schizophrenia and other psychiatric disorders, in particular bipolar disorder (17–19). As current findings explain a modest proportion of total schizophrenia susceptibility, expanding the number of risk mutations will be important in understanding molecular etiology but also the relationships between these clinical disorders (20).

We report a gene-based analysis of large (>100 kb), rare [<1% minor allele frequency (MAF)] CNVs in the Wellcome Trust Case Control Consortium 2 (WTCCC2) schizophrenia sample of 1564 cases and 1748 controls, all from Ireland. We find evidence of association with duplications at 20p12.2 and further support in a large independent European sample. All of the carriers were from the British Isles and an extended analysis including 32 911 Irish/UK subjects provided further association support. By haplotype analysis and sequencing, we show that the CNV is not the result of repeated *de novo* events and is an inherited risk mutation potentially inherited from a single European ancestor. This accords with emerging evidence for rare CNVs that are population-specific and associated with substantial illness risk (21,22), consistent with the ‘Clan Genomics’ concept (23). The mutation involves a tandem duplication of 148 951 bp at chr20:9,684,767–9,833,717(hg18) overlapping the gene p21 *Protein-Activated Kinase 7* (*PAK7*, also known as *PAK5*). Other PAK family members modulate synaptic network development through a signaling pathway regulated by the schizophrenia risk gene *DISC1* (24). We demonstrate that *PAK7* is co-expressed with *DISC1* in developing brain and further investigation of the role of the mutation in synaptic mechanisms salient to schizophrenia is warranted.

## RESULTS

### Discovery phase evidence of association at four loci

We observed evidence of association (*P* < 0.05) at four loci in the Irish discovery dataset of 1564 cases and 1748 controls; 20p12.2(*PAK7* [MIM 608038]); 2cen-q13 (*ANKRD36B, COX5B* [MIM 123866]; *ACTR1B* [MIM 605144]); 3p25.1 (*MRPS25* [MIM 611987], *ZFYVE20* [MIM 609511]) and a previously confirmed schizophrenia risk locus [chr1q.21 (*CHD1L* [MIM 613039]) locus (8,9)]. Association results for the three novel loci are presented in Table 1. To improve our estimate of the population frequency of what are rare events, we examined the three novel loci in an extended control sample (*n* = 6944) including UK controls from WTCCC2 [2533 controls from the UK National Blood Service (NBS) and 2663 from the UK 1958 Birth Cohort (58BC)]. We identified chr1q21.1 risk duplications in four cases, chromosome 15q13.3 deletions in three cases and a single case with the large 22q11.2 deletion with no similar events seen in controls from the discovery analysis. Details of our findings at all previously reported schizophrenia CNV risk loci, with locus coordinates, are provided in Supplementary Material, Table S3a. Further, we provide details of all large (>100 kb), rare CNVs identified in our discovery sample in cases and controls (Supplementary Material, Table S3b).

**Table 1.** Association analysis of large duplications at three loci in psychosis case–control samples

Locus	Discovery					Replication 1			Replication 2			Combined analysis		
Chr Position Gene(s)	Irish SZ cases (*n* = 1564)	Irish controls (*n* = 1748)	*P*-value^a^	UK 58C + NBS controls (*n* = 5196)	*P*-value^a^	ISC SZ cases (*n* = 3111)	ISC controls (*n* = 2267)	*P*-value^b^	BPD cases (*n* = 2243)	UK controls (*n* = 10 769)	*P*-value^b^	Irish and UK cases (*n* = 11 707)^c^ (including 6223 CLOZUK samples)	Irish and UK controls (*n* = 21 204)^d^ (including 7703 WTCCC2 controls)	*P*-value^b^ OR (95% CI)
20p12.2, PAK7	5	0	0.023	3	0.007	6	0	0.052	4	5	0.047	22	10	0.0002, 11.3, (3.7, ∞)
2cen-q13, COX5B ANKRD36B ACTRIB	12	1	0.001	9	0.0001	3	2	NS						
3p25.1, ZFYVE20 MRPS25	6	0	0.011	1	0.0002	0	1	NS						

^a^Fisher's exact test (two-tailed *P*-value).

^b^One-sided CMH test used for replication and combined analyses due to multisite nature of samples. NS, non-significant (*P* > 0.1).

^c^Excludes non-Irish and non-UK cases (*n* = 1434) and controls (*n* = 1283) from the ISC Replication 1 sample.

^d^Includes 2507 additional UK controls from the People of the British Isles (POBI study) with two PAK7 duplication carriers which were unavailable at the time of discovery analysis.

### Replication phase association evidence at chr20p12.2

Three loci were carried forward for replication (Table 1) in the independent International Schizophrenia Consortium (ISC) dataset of 3111 cases and 2267 controls from the UK, Portugal, Sweden and Bulgaria (8). Only the chr20p12.2 locus containing p21 *Protein-Activated Kinase 7* (*PAK7*, also known as *PAK5*) showed evidence for association in the replication sample, with six duplication events, all in UK cases, and none in controls (*P* = 0.052). Because this is a rare event, and absent in the control population, the reported association result was the most significant *P*-value obtainable without a larger sample size.

As all of the carriers were identified in samples from the British Isles, we investigated the locus for additional evidence of association in an independent sample of bipolar disorder in the WTCCC1 (*n* = 1697) (25) and University College London (UCL) (*n* = 546) samples (26). The controls included independent UK controls from the UCL study (*n* = 510) and 10 259 cases ascertained for nonpsychiatric disorders in WTCCC1. We identified four duplication carriers in cases and five carriers in the control sample, provided further nominal evidence of association (*P* = 0.047).

We performed a combined analysis of all Irish/UK subjects including additional psychosis cases from the CLOZUK study (27) (*n* = 6223) and independent controls from the WTCCC2 UK control sample (*n* = 7703) (28,29). Combining the evidence across studies, under the assumption of the same effect on risk in each strata, gave a meta-analysis Cochran–Mantel–Haenszel (CMH) *P*-value = 2 × 10^−4^ (OR = 11.3, 95% CI = 3.7, ∞) with 22 carriers in 11 707 psychosis cases and 10 carriers in 21 204 controls.

### Evidence for a single founder risk mutation at PAK7

**Figure 1.**
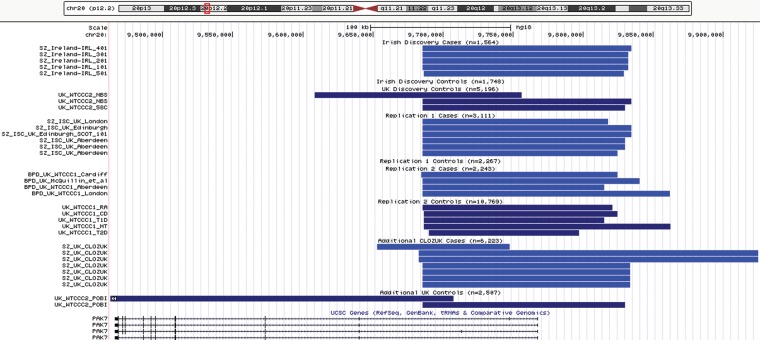
Inferred position of duplication events at the chr20p12.2 locus in build hg18. SZ, schizophrenia. BD, bipolar disorder. See Supplementary Material, Table S5 for exact start/stop coordinates. Duplications in controls are colored light blue and SZ or BP cases are colored dark blue.

Based on the array data, we examined the inferred breakpoints for the chr20p12.2 duplications, and the estimated start and stop coordinates for the 32 duplication carriers (Fig. 1, Supplementary Material, Table S5). Nineteen of the 22 duplication carriers in the cases and 8 of 10 carriers from the controls exhibited duplications of 132–146.5 kb with very similar start positions (∼9.68 Mb), apparently similar breakpoints across samples and no evidence of flanked segmental duplications. The evidence for association at the locus in the Irish/UK sample appeared to be driven by these duplication cases. The remaining five individuals carried duplications occurring on different haplotype backgrounds and are likely to represent different molecular events.

To explore whether this could represent a single ancestral mutation event, we examined haplotype sharing in the five Irish case samples in linkage disequilibrium (LD) blocks (termed Hap Block 1 and Hap Block 2) immediately 5′ and 3′ of the inferred duplication. Haplotypes were phased in PLINK with a probability >0.98. All five Irish cases carried a copy of the same haplotype in both Hap Block 1 (CCTT, *f* = 0.188) and Hap Block 2 (TCAA, *f* = 0.208) (Supplementary Material, Tables S6 and S7). We extended the analysis to include 18 of the 22 UK carriers (as these had been genotyped on the same Affymetrix platform) and found that all carried copies of the CCTT and TCAA haplotypes flanking the duplication (Supplementary Material, Table S8). Given the low level of LD between these blocks (*D′* = 0.22, *r^2^* = 0.02) the probability of making these observations if the duplication was the result of repeated *de novo* mutations is extremely low (*P* = 2.5 × 10^−21^), providing support for a single ancestral duplication event. We did not find an increased level of relatedness among the Irish *PAK7* duplication carriers compared with the level of relatedness of random Irish individuals, arguing against a very recent event (see Supplementary Material, Fig. S1).

**Figure 2.**
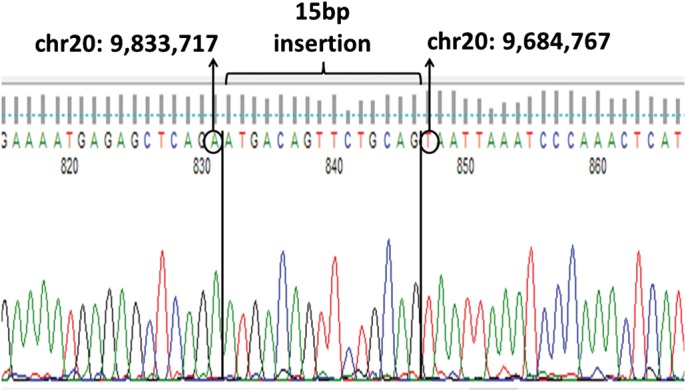
Capillary sequence of the unique region in the middle of the two copies of the tandem repeat sequence at the *PAK7* duplication. The first copy of the sequence ends at chr20:9,833,717(hg18) and the second copy starts with sequence from chr20:9,684,767(hg18), thereby defining the breakpoints. A 15 bp sequence is present between the two copies.

Next, we confirmed the duplication event in one individual (IRL_101) using an Agilent custom designed comparative genomic hybridization (CGH) microarray with high probe density in the *PAK7* region. The breakpoints identified by CGH (chr20:9,684,902–9,833,151) map closely to those predicted by the SNP array analyses (chr20:9,685,413–9,831,947; Supplementary Material, Fig. S2).The event overlaps exon 1 and enhancer and promoter regions adjacent to exon 1 in all four known *PAK7* transcripts, and exon 2 in two alternative transcripts and is absent from the Database of Genomic Variants (http://projects.tcag.ca/variation/). By capillary sequencing this individual, we identified a tandem duplication of 148 951 bp at chr20:9,684,767–9,833,717(hg18) with a 15 bp sequence inserted between the first and second copies of the repeated sequence (Fig. 2). By sequencing the five remaining Irish carriers and two of the UK carriers (SCOT_101, SCOT_201), we confirmed that all shared exactly the same breakpoints with the same 15 bp sequence inserted between the two copies of the repeat sequence, suggesting that this represents the same duplication event.

### Assessment of additional family members of carriers

**Figure 3.**
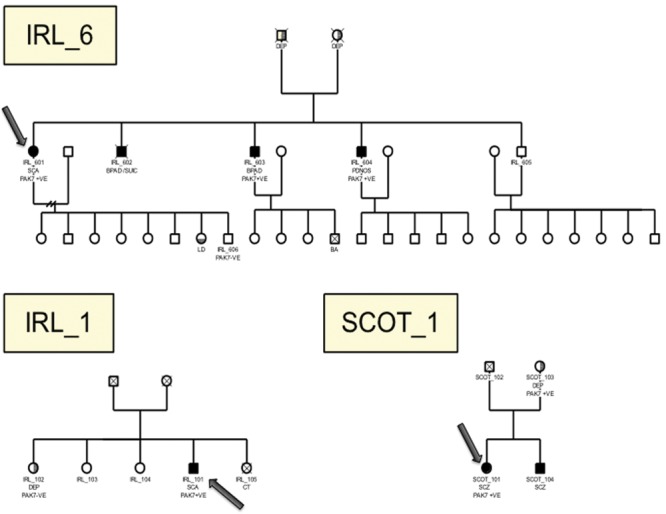
Familial pedigrees for patients from three families (IRL_6, IRL_1, SCOT_1) who were found to be positive for *PAK7* duplications. The index individual in each family screened is indicated with an arrow. Individuals positive (PAK7 + VE) or negative (PAK7 − VE) for PAK7 are indicated. Filled symbols indicate individuals with psychotic disorders (SCZ, schizophrenia; SCA, schizoaffective disorder; BPA, bipolar affective disorder; PDNOS, psychotic disorder not otherwise specified). Partial vertically shaded symbol indicates depressive disorder (DEP, depression). Partial horizontal shaded symbol indicates learning disability (LD). Cause of death where known: SUIC, suicide; BA, birth asphyxia; CT, cerebral tumor.

We were able to access additional family members of two cases to test for evidence of co-segregation of the duplication with illness (see Fig. 3). In the family of IRL-101, there were three other surviving siblings, none of whom had a diagnosis of schizophrenia or a related major mental disorder. One sister (IRL_102) agreed to be tested. She had experienced a depressive episode after bereavement and did not carry the duplication. By going back to the original GWAS data, we identified another Irish case (IRL_601) that had been excluded from the primary analysis because they are related to IRL_101 based on a proportion of identity-by descent estimated at 0.033. IRL_601 is a female patient with a history of Schizoaffective Disorder. She is the youngest in a sibship of five children of which four were affected with a major psychotic disorder. We confirmed that two affected brothers (IRL_603 (bipolar I disorder), IRL_604 (psychotic disorder, not otherwise specified)) also carry the *PAK7* duplication. The remaining affected brother [IRL_602 (bipolar I disorder)] died by suicide. We tested one of the eight offspring of IRL_601, and this unaffected individual did not carry the duplication. In the SCOT_1 family, we confirmed that the duplication was inherited from the mother (SCOT_103). Neither of the parents was affected clinically, but there was a family history of depression in both families, and a brother with a history of psychosis did not carry the duplication (SCOT_104).

### Is the mutation present in other European ancestry populations?

To assess whether the mutation was exclusive to the British Isles or present elsewhere in Europe, we examined additional European populations. Details of the cohorts analyzed are included in Supplementary Material, Table S1. The estimated control frequency for the mutation in the UK/Irish population is low (*f* = 0.037%) and most of these cohorts were underpowered to provide an accurate frequency estimate for what is a rare event. We failed to identify any carriers in large Icelandic (>72 000 individuals) and Finnish samples (∼3800 individuals), but did find carriers in populations from the Eastern USA, the Netherlands, Sweden and Denmark. We were able to unambiguously phase haplotypes in five US control carriers with available Affymetrix data, confirming that all of these individuals shared the same haplotype background as the Irish/UK cases, suggesting that they carry the same mutational event although the samples were not available for confirmatory sequencing. The other European samples were genotyped on a different array platform and where we could definitively phase haplotypes; this confirmed the same haplotype background. These data suggest that the mutation is present in other European populations. The limited number of cases available precluded formal association analyses, but we identified four additional cases with the duplication in Dutch, Danish and Swedish cohorts. Information on the clinical characteristics, illness course, co-morbidity and family history for all mutation case carriers is provided in Supplementary Material, Table S9.

### Functional investigation of PAK7

The p21-activated kinases (PAKs) are a family of serine/threonine protein kinases, which are regulated by the Rho family of small G proteins and are involved in multiple intracellular signaling pathways. Six PAK genes are expressed in human and based on their regulatory functions are classified into Group I (*PAK 1-3*) and Group II (*PAK4-6*) members (30). Group I PAKs are activated by RAC-PAK signaling to promote axon connectivity, and synapse formation, in the developing brain in a pathway regulated by another schizophrenia risk gene *DISC1* [MIM:60521] (31). Aberrant synaptic network development and maintenance represents a plausible molecular mechanism for psychosis susceptibility and mutations involving other Group I PAKs (*PAK2* (the 3q29 microdeletion syndrome locus [MIM 609425]) and *PAK3* [MIM:300558]) are known to be associated with neurodevelopmental syndromes characterized by psychosis (32). *PAK7* is a brain-specific isoform within the cell it is localized to filipodia, where it has been shown to promote the induction of neurite outgrowth, filopodium formation and synaptic vesicle trafficking (33). The Group II PAKs, including *PAK7*, differ structurally from Group I members and their mechanism of activation requires clarification.

**Figure 4.**
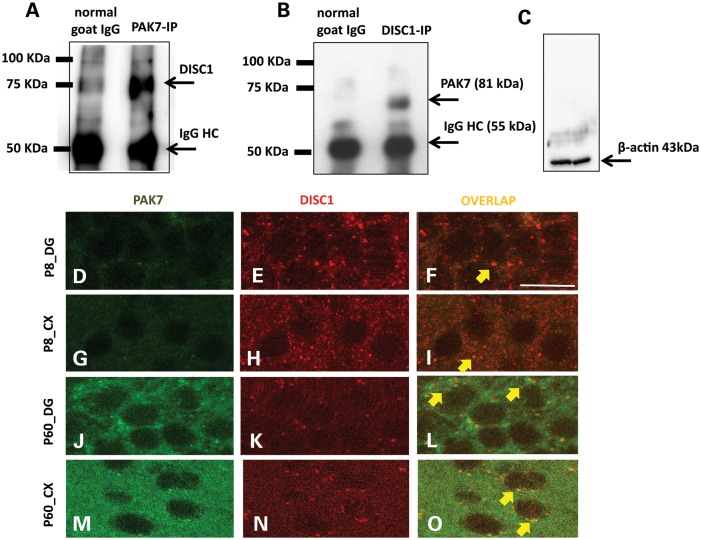
PAK7 and DISC1 interact in mouse brain synapses. (**A**) Immunoprecipitation of PAK7 and DISC1 from synaptoneurosomal extracts of adult mouse brains. The extract was immunoprecipitated for PAK7 and the eluate was immunostained for DISC1. (**B**) Reciprocal experiment described in A: immunoprecipitation of DISC1 followed by immunostaining of the eluate with antiPAK7. In both A and B, the samples immunoprecipitated with control IgG showed no signal, showing the specificity of the PAK7–DISC1 interaction. An additional control in (**C**) shows that the brain extracts before immunoprecipitation have the same amount of loading control (β-actin). (**D**–**N**) Immunostaining for PAK7 (green) and DISC1 (red) in brain sections containing Hippocampus and Cortex of P7 (D–I) and Adult (J–**O**) mice. The experiment shows an overlapping (yellow) of PAK7 and DISC1 in puncta, confirming a synaptic interaction between the two proteins. The co-localization is particularly evident in the adult (J–O) preparation.

To test whether *PAK7* may be under similar regulatory control to Group I PAKs during early brain development, we investigated the relationship between *PAK7* and *DISC1*. Examining Brainspan data (brainspan.org), we identify significant negative correlation between DISC1 and PAK7 expression in whole human brain throughout life (*r*^2^ = −0.297 to 0.867). The strongest correlations are for the first and last trimester of pregnancy and the first 5 years of life, with the level of correlation falling through adulthood. In a co-immunoprecipitation experiment, we confirmed interaction between *PAK7* and *DISC1* in synaptoneurosomal preparations from full mouse brain at postnatal day 8–10 [(P8–10); Fig. 4A]. Next, we ran the reciprocal experiment and demonstrated immunoprecipitation of DISC1 followed by immunostaining of the eluate with antiPAK7 (Fig. 4B). Finally, we explored the regional and temporal evidence for co-expression in brain regions implicated in schizophrenia etiology (hippocampus and cortex). In this analysis, we co-immunostained PAK7 and DISC1 in P7 and adult (>P60) mice. We find clear overlap in perisomal puncta, which confirms that the two proteins interact at the synapse, particularly in adult mouse brain (Fig. 4D–O). This suggests that *PAK7* is developmentally co-expressed with *DISC1* and may have a specific functional role at the synapse.

## DISCUSSION

We report evidence that rare, chr20p12.2 duplications increase risk for the major psychotic disorders, schizophrenia and bipolar disorder. Previous schizophrenia studies have identified at least eight rare CNVs of strong effect (genotypic relative risks of 4–20) (5). Most of these CNVs have been identified because they recur in the population, either as relatively large events implicating many genes and arising from nonallelic homologous recombination (NAHR), or through the accumulation of different, generally *de novo* mutations at a single locus (e.g. *NRXN1*, *VIPR2*). At the *PAK7* locus, we identified a single founder event with a 15 bp sequence inserted between the two copies of the tandem duplication. From the 1000 Genomes Project data, most tandem duplications of this type are formed by fork stalling and template switching as detailed in the micro-homology-mediated break-induced replication model (34,35).

We identified 2653 genes affected by a CNV (>100 kb but <10 Mb) in at least one individual in our study. However, only 382 genes had the four or more overlapping CNVs required to achieve *P* < 0.05 in our discovery sample. Based on our data, the overall contribution of CNVs to psychosis susceptibility risk is difficult to estimate as we could not exclude rarer or smaller events. Ongoing CNV analysis of the Psychiatric Genomics Consortium dataset will be important in more comprehensively investigating rare, large CNV events (17).

By haplotype analysis and sequencing, we established that the association is driven by a ∼149 kb duplication at the gene *PAK7* with a likely common European ancestor. Inherited mutations have been described contributing to other neurological disorders, but their relative contribution to schizophrenia susceptibility is unknown (21,22,36). Such events are likely to be recent in origin and differentiated between populations making them difficult to replicate across diverse collections using standard gene association methods in large multicohort CNV studies (37–39). Although a single variant is unlikely to influence the overall population risk for a disorder, founder risk mutations can be identified through association analysis in specific populations. By examining cases excluded, through relatedness QC, from our original Irish GWAS dataset, we identified an additional duplication carrier related at a distance of five meioses to one of the index cases. Within the family of this additional carrier, we found evidence of co-segregation of the duplication with psychosis in two affected siblings. Identifying cryptic relationships between individuals in available GWAS datasets may be helpful in identifying extended pedigrees for analysis as a complementary approach to existing association methods.

The risk duplication identified in this study overlaps the first two exons and enhancer and promoters regions in the known transcripts of *PAK7*. Because *PAK7* is exclusively brain expressed, the impact of the duplication on gene expression and the genetic mechanism involved are yet to be determined. This is important as we identified other larger duplication events at the locus in two control samples, but do not know if these would have the same or different effects on gene function. From existing animal models of *PAK7*, we do know that knockout mice are viable with no obvious developmental abnormalities but *PAK6/PAK7* double knockout mice show behavioral and learning deficits suggesting functional redundancy between these isoforms (40). Genomic studies in carriers of the founder mutation will be important as additional risk variants or mutations (e.g. in *DISC1* or *PAK6*) may be contributing to risk in this extended pedigree.

The *PAK7* risk mutation is rare, but based on data from the British Isles, we estimate the exposed attributable risk for carriers is likely to be substantial (41). We attempted to date the origin of the duplication by examining haplotype sharing in this genomic region. After characterizing the haplotype patterns in the region, we were unable to make robust inference: further family data will be required to accurately phase haplotypes for this rare event.

Almost all of the risk CNVs identified to date in the schizophrenia literature also increase susceptibility to other developmental phenotypes including intellectual disability, autism and seizure disorder. Only four of our cases received a full cognitive assessment (including IQ measurement), and all were assessed as being within the normal range of cognitive function. One individual had a history of co-morbid language delay and another had a history of seizure disorder. Although most of the cases had schizophrenia, seven had bipolar disorder or schizoaffective disorder. This is in keeping with evidence of shared genetic etiology between these disorders, although the evidence for CNV involvement in bipolar disorder has until now been more equivocal (42,43). Having identified carriers of the duplication in control cohorts, we were interested in testing whether ‘control’ individuals had psychiatric or developmental phenotypes. In almost all studies (including the individual WTCCC1 studies), controls were not screened for mental disorder and little if any developmental information was available. Within the UK and Ireland health biobank, data are now becoming available on a sufficiently large scale to allow us to directly examine the phenotypic implications of carrying rare events in the general population and this is one of future goals (e.g. The Generation Scotland Family Health Study, UK Biobank and ‘Growing Up in Ireland’ Study). Further clinical and genetic assessment of patient carriers and their families will also be important in quantifying the penetrance and range of phenotypic expression of the *PAK7* duplication.

Schizophrenia is a neurodevelopmental disorder and a disease model involving aberrant synaptic network development has been proposed (44). Group I PAKs play a role in the development of these networks through a signaling pathway regulated by an established susceptibility gene (DISC1) (45). *PAK7* is a brain-specific isoform, within the cell it is localized to filipodia, where it has been shown to promote the induction of neurite outgrowth, filopodium formation and synaptic vesicle trafficking (33). To clarify its mechanism of activation, we performed functional studies that confirmed interaction between *PAK7* and *DISC1*, suggesting that this gene may be under similar regulatory control to other PAK family members. Furthermore, this interaction occurs at the synapse and is particularly evidence in adult mouse brain suggesting a role in synaptic plasticity.

In conclusion, we have identified a psychosis risk duplication at the gene *PAK7*, with evidence that it is inherited from a common ancestor. The gene functions in the development and maintenance of synaptic networks and may be under the regulatory control of *DISC1*. Many of the cases segregating the DISC1 translocation in the original Scottish schizophrenia pedigree were affected with schizoaffective disorder or mood disorders as was the case in the largest *PAK7* pedigree we investigated, and we also found evidence for association with bipolar disorder (46). This suggests a broader molecular risk mechanism for psychosis. A critical next step will be to understand how the duplication impacts on gene expression and function so that it can be investigated in model systems. As *PAK7* is exclusively brain-expressed, this will require further experimental work.

## MATERIALS AND METHODS

### Study subjects

The discovery sample included 1564 cases and 1748 controls from the Irish Schizophrenia Genomics Consortium/WTCCC2 GWAS study which has previously been described (47). Participants, from the Republic of Ireland or Northern Ireland, were interviewed using a structured clinical interview and diagnosis of schizophrenia (*n* = 1418) or a related disorder [schizoaffective disorder (*n* = 182); schizophreniform disorder (*n* = 6)] was made by the consensus lifetime best estimate method using DSM-IV criteria. Control subjects were ascertained with written informed consent from the Irish GeneBank and represented blood donors from the Irish Blood Transfusion Service. Cases and controls met the same ethnicity criteria (Irish origin with all four grandparents born in Ireland or the UK). To improve our estimate of the population frequency of what are rare events, we examined nominally significant loci in an extended control sample (*n* = 6944) including UK controls from WTCCC2 [2533 controls from the UK National Blood Service (NBS) and 2663 from the UK 1958 Birth Cohort (58BC)] (28).

The first phase of replication examined nonoverlapping subjects from the ISC dataset (8). To follow-up evidence of association in the British Isles, we performed further association analysis in bipolar disorder cases and controls from the UK/Ireland. The WTCCC1 bipolar disorder sample included 1697 cases recruited throughout the UK. Most of the cases had a diagnosis of bipolar I disorder (71%) or schizoaffective disorder (15%). Controls were nonpsychiatric disease case participants in the WTCCC1 study (*n* = 10 259) (25). The UCL bipolar disorder sample included 546 individuals (97% bipolar I disorder) and comparison subjects (*n* = 510) with no personal or first-degree history of any mental disorder and from a similar UK or Irish ancestry, based on the origin of all four grandparents (26).

Finally, in an extended analysis, we included additional psychosis cases and controls from the UK. The additional psychosis cases were of Caucasian origin and came from the CLOZUK sample (*n* = 6223) (27). The CLOZUK sample consists of patients taking the antipsychotic medication Clozapine, a drug reserved for the treatment-resistant psychosis patients in the UK. The remaining control subjects were ascertained as described in previous WTCCC2 studies from the UK National Blood Service (NBS) (*n* = 2533); the UK 1958 Birth Cohort (*n* = 2663); the People of the British Isles (POBI) study and from (28,29). We also investigated additional European ancestry cohorts for evidence of the PAK7 duplication and details on the samples tested, case and/or control numbers and genotyping platform are provided in Supplementary Material, Table S1 (5,9,48,49).

### CNV calling and validation

We report a schizophrenia study investigating large (>100 kb), rare (<1% MAF in all samples) CNVs using data from the WTCCC2. All discovery samples were genotyped using the Affymetrix 6.0 platform either at the Affymetrix (Santa Clara, CA, USA) or at the Broad Institute (Cambridge, MA, USA) laboratories. Samples were processed using the WTCCC2 pipeline and quality control details have been reported previously (27,47).

CNV calls were created using Birdseye from Birdsuite (version 1.5.5) (50) for autosomes only, and we excluded calls where lengths were <100 kb or >10 Mb, or LOD score <10. We excluded CNVs with at least 50% overlap with a region copy-number variable in at least 1% of samples; individuals with >30 CNV calls or a total event length >10 Mb and calls for samples from plates containing fewer than 40 samples (Supplementary Material, Table S2). Calls with a copy number of 0 or 1 were considered to be deletions and calls with a copy number of 3 or 4 were considered duplications.

For the replication analysis, the ISC data were called using the same calling protocol as in the discovery analysis. Details on genotyping platforms used for the other samples are provided in Supplementary Material, Table S1. For the identified *PAK7* risk duplication, we performed standard qPCR validation of the CNV calls in the discovery sample (Supplementary Material, Table S4). Verifying the presence of CNVs by this method addresses sensitivity, but not the specificity of CNV calling. To test the specificity and sensitivity of calling across genotyping platforms, we examined probe intensity data for 6542 control subjects who had been genotyped on Affymetrix 6.0 and Illumina 1.2M-Duo arrays as part of the wider WTCCC2 study. We called CNVs on the Illumina platform using QuantiSNP (51) and excluded those with log Bayes factor <10, length <100 kb or >10 Mb. We excluded samples with more than 10 CNVs in total, with more than 10 Mb of total CNV length, or failed SNP quality control. This identified the same four individuals as the only PAK7 duplication carriers across different arrays and calling algorithms. For the additional European ancestry cohorts, CNV calls were made as described in the primary publications [see Supplementary Material, Table S1 and references (5,9,48,49)]. The CLOZUK calling method is described in Guha *et al*. (2013) (52).

### CNV analysis

We conducted a gene-based analysis using gene boundaries from UCSC Genome Browser refGene (hg18) and identified 2653 genes impacted (from the transcription start to end point) by a CNV (>100 kb but <10 Mb) in at least one individual. Three hundred eighty-two genes had at least four overlapping CNVs (required to achieve *P* < 0.05) in the discovery data, representing a smaller number of loci, as some CNVs overlap multiple genes. Fisher's exact tests were used to calculate *P*-values in the discovery analysis and a fixed-effects CMH test to assess the evidence across replication cohorts.

### Haplotype analysis

We analyzed carriers of the ‘common’ *PAK7* duplication at chr20:9,685,413–9,831,947, which is carried by 27 of 32 individuals with duplications overlapping the *PAK7* locus in the British Isles samples. Haplotype analysis used a core set of Affymetrix SNPs that had been genotyped in 23 of the samples and all genotype data were converted to the forward strand. The four samples omitted were from the CLOZUK sample genotyped on Illumina where no suitable proxy SNPs were available for analysis. From HapMap data, the start of the duplication sits in a haplotype block that extends from rs2423462 (9682770) to rs2423467 (9689876). The part of this block that is outside the region, termed Hap Block 1 is from rs2423462 (9682770) to rs742450 (9684493). The duplication ends in a region between haplotype blocks. Immediately, 3′ of the duplication is a haplotype block that extends from rs6057009 (9840327) to rs6118819 (9867572) (Hap Block 2). We have focused on haplotypes outside the duplication region, to analyze diploid genotypes, and limited the analysis to regions of high LD where haplotypes could be phased. In the absence of family data, extended haplotypes outside these local blocks could not be accurately phased. Haplotype frequencies and phased haplotypes, with a probability >0.98, were estimated using the –hap and –hap-phase functions in Plink (see Supplementary Material, Tables S6 and S7). For either Hap Block, the chances that all 23 samples would carry at least one copy of a specific haplotype, i.e. be either heterozygous or homozygous for this haplotype *q* = [*q*^2^ (probability of homozygous *q*) + 2*q*(1 − *q*) (probability of homozygous *q*)]^23^ = (2*q* − *q*^2^)^23^ where *q* is the haplotype frequency. This probability was calculated for both Hap Blocks and in the absence of LD between the blocks, these numbers were multiplied together to determine the combined probability of all 23 carriers carry copies of the same haplotypes either side of the duplication.

### CNV breakpoint sequencing

Using an Agilent custom designed CGH microarray with high probe density in the *PAK7* region, we confirmed the duplication event in one individual and the breakpoints identified by CGH (chr20:9,684,902–9,833,151) map closely to those predicted by the SNP array analyses (chr20:9,685,413–9,831,947; Supplementary Material, Fig. S4). Based on these estimated breakpoints and assuming that this is a tandem duplication that is not inverted, we attempted to sequence from one copy of the duplication into the second copy in order to identify the precise breakpoints. Using a forward primer positioned at the end of the duplication (TCTCTGTTGGATGGAGCTTCT) and a reverse primer positioned at the start of the duplication (CGATGTAAAAAGACACAAGAGAAA), we successfully PCR-amplified this unique region in a carrier sample. The PCR did not amplify in noncarrier samples. Using capillary sequencing, we identified the event as being a tandem duplication of 148 951 bp at chr20:9,684,767–9,833,717(hg18) with a 15 bp sequence inserted between the first and second copies of the repeated sequence (Fig. 2).

### Functional analysis

#### Protein extraction

Synaptoneurosome preparation was performed from full mouse brain at P8–10 as described elsewhere with modifications (53). Briefly, brain tissue was glass/glass homogenized in ice-cold lysis buffer daily-fresh made until complete lysis. The protein extraction buffer contained 10 mm Tris Base, 0.1m sodium chloride, 4 mm EDTA, 0.1% NP40 and was added with protease inhibitor cocktail (Roche Diagnostic). The homogenate was centrifuged at 1000*g* × 10 min at 4°C in order to separate heavy cellular membranes. The resulting supernatant was passed through two 105 µm polypropylene mesh (Amazon, USA) and a 5 µm nitrocellulose filter (Millipore) and finally centrifuged at 1000 g × 15 min at 4°C. The obtained synaptoneurosomal-depleted supernatant contains cytoplasmic proteins and was collected in fresh tubes; while the pellet, which is enriched in synaptoneurosomal proteins, was resuspended in protein extraction buffer. Protein concentration was measured by spectrophotometer quantification (Nanodrop, ND8000) in both cytoplasmic and synaptoneurosomal-enriched extracts for further analysis.

Western blot was carried out by standard procedures with the following specifications: protein samples were fractioned through 8 or 10% acrylamide gel and blotted onto PVDF membrane. The membrane was probed overnight with antibody anti-MAP2 (mouse monoclonal, Millipore, MAB3418, 1:2500) and antibody anti-PSD95 (rabbit polyclonal, Cell Signaling Technology, 2507, 1:2500). Antibody anti-β-actin (rabbit polyclonal, Cell Signaling Technology, 4967, 1:5000) was used as a loading control. After 16 h, three washes of 5 min with 0.5% Tween/TBS were carried out followed by 1 h incubation with the following secondary antibodies: anti-mouse HRP-linked IgG (Cell Signaling Technology, 7076, 1:10 000) and anti-rabbit HRP-linked IgG (Cell Signaling Technology, 7074, 1:10 000). After washing as above, ECL substrate (Millipore) was applied and chemiluminescent signal was revealed by exposing photographic film (Sigma, Z370398) to the membrane for ∼5 min.

#### Co-immunoprecipitation

Co-immunoprecipitation procedure was adapted from Felli *et al*. (2005) (54) as described below. Synaptoneurosomal protein extracts were made from full brain tissue as described and PAK7 was immunoprecipitated. For both experiments, 500 µl-volume samples containing 1000 µg of synaptoneurosomal extracts were obtained by dilution in protein extraction buffer. Samples were immunoprecipitated with either 4 µg antibody anti-PAK7 (polyclonal goat, S-16, Santa Cruz Biotechnology, sc-22155) or 4 µg antibody anti-DISC1 (polyclonal goat, N-16, Santa Cruz Biotechnology, sc-47990). Normal goat IgGs (Sigma, I5256) were used as a negative control. After 16 h, all samples were incubated with 25 µl of beads (protein A/G PLUS-Agarose, Santa Cruz Biotechnology, sc-2003) for 4 h and washed. Samples were then resolved by western blotting as described, and probed with antibody anti-DISC1 (rabbit polyclonal antibody anti-DISC1 Mid, Invitrogen, 40–6900, 1:1500) or anti-PAK7 (polyclonal rabbit anti-PAK7, Sigma, SAB3500335, 1:1000). Primary antibody incubation was followed by incubation with biotinylated anti-rabbit IgG (Vector Labs, BA-1000, 1:3000) and amplification with Vectastain ABC kit (Vector Labs, PK-6100).

#### Free floating immunohistochemistry

For histological staining, brain tissue from mice at three different developmental stages was used: P7 (postnatal day 7) and *P* > 60. The brains, previously fixed in 4%paraformaldehyde/PBS and kept in 20%sucrose/PBS at 4°C, were sliced using a vibratome (Leica) to obtain 50 µm-thick coronal sections. After 5 min incubation with 0.1% hydrogen peroxide, sections were washed in PBS, and moved for 1 h into a PBS blocking buffer containing 10% goat serum and 3% Triton for tissue permeabilization. Sections were then incubated for ∼36 h in blocking buffer added with the following primary antibodies: anti-PAK7 (polyclonal goat, Santa Cruz Biotechnology, sc-22155, 1:750), anti-DISC1 (polyclonal rabbit, Invitrogen, 40–6900, 1:500), anti-Synapsin 1/2 (polyclonal guinea pig, Synaptic Systems, 106 004, 1:1000). Primary antibody probing was followed by several PBS washes for a total time of 2 h. Sections were then moved to a secondary antibodies solution made of 0.05%Triton-X100 and added with the following fluorofore-conjugated antibodies: DyLight 488 donkey anti-goat IgG (Jackson ImmunoResearch Labs, 705-485-003, 1:500), DyLight 649 goat anti-rabbit IgG (Jackson ImmunoResearch Labs, 111-495-003, 1:500) and Cy3 goat anti-guinea pig IgG (Jackson ImmunoResearch Labs, 106-165-003, 1:500). After 2 h, sections were washed for 15 min in PBS and mounted onto microscope slides using media containing DAPI (Vector H-1200). Co-immunostaining of PAK7 and DISC1 was confirmed by confocal microscope imaging (Zeiss, ×63 objective) in hippocampus and brain cortex fields at each considered age.

## SUPPLEMENTARY MATERIAL

Supplementary Material is available at *HMG* online.

## FUNDING

Funding for this study was provided by the Wellcome Trust Case Control Consortium 2 project (085475/B/08/Z and 085475/Z/08/Z), the Wellcome Trust (072894/Z/03/Z, 090532/Z/09/Z and 075491/Z/04/B), NIMH grants (MH 41953 and MH083094) and Science Foundation Ireland (08/IN.1/B1916). We acknowledge use of the Trinity Biobank sample from the Irish Blood Transfusion Service; the Trinity Centre for High Performance Computing; British 1958 Birth Cohort DNA collection funded by the Medical Research Council (G0000934) and the Wellcome Trust (068545/Z/02) and of the UK National Blood Service controls funded by the Wellcome Trust. Chris Spencer is supported by a Wellcome Trust Career Development Fellowship (097364/Z/11/Z). Funding to pay the Open Access publication charges for this article was provided by the Wellcome Trust.

## Supplementary Material

Supplementary Data
