# Supplementary material for: An inherited duplication at the gene p21 Protein-Activated Kinase 7 (PAK7) is a risk factor for psychosis
Source: Hum Mol Genet. 2014 Jan 28;23(12):3316–26. doi: 10.1093/hmg/ddu025 (PMC4030770; doi:10.1093/hmg/ddu025)
Supplement: Supplementary Data [file supp_23_12_3316__index.html]

An inherited duplication at the gene p21 Protein-Activated Kinase 7 (PAK7) is a risk factor for psychosis — An inherited duplication at the gene p21 Protein-Activated Kinase 7 (PAK7) is a risk factor for psychosis — An inherited duplication at the gene p21 Protein-Activated Kinase 7 (PAK7) is a risk factor for psychosis — Supplementary Data 

# An inherited duplication at the gene *p21 Protein-Activated Kinase 7* (*PAK7*) is a risk factor for psychosis

## Supplementary Data

Supplementary Data

**Files in this Data Supplement:**

- Supplementary Data - Docx file
- Supplementary Figure 1 - jpg file
- Supplementary Figure 2 - jpg file
- Supplementary Figure 3 - jpg file
- Supplementary Figure 4 - jpg file
- Supplementary Tables - xls file
