# Supplementary material for: An inherited duplication at the gene p21 Protein-Activated Kinase 7 (PAK7) is a risk factor for psychosis
Source: Hum Mol Genet. 2014 Jan 28;23(12):3316–26. doi: 10.1093/hmg/ddu025 (PMC4030770; doi:10.1093/hmg/ddu025)
Supplement: Supplementary Data [file supp_ddu025_ddu025supp.docx]

**Supplementary material**

**An inherited duplication at the gene *p21 Protein-Activated Kinase 7 (PAK7)* is a risk factor for psychosis.**

**SUPPLEMENTARY INFORMATION**

**Table S1: Details of all samples in the study**

**Table S2: Sample exclusion details for Discovery sample**

**Table S3A & B: Previously reported associated CNVs and all CNVs in discovery**

**Table S4: Results of qPCR validation**

**Table S5: Start and stop coordinates for *PAK7* duplication in carriers**

**Table S6: SNP haplotypes at *PAK7* locus**

**Table S7: Haplotype frequencies and phased haplotypes estimation**

**Table S8: Phased haplotypes for each block at *PAK7* locus**

**Table S9: Clinical details and family history in *PAK7* carriers**

**Supplementary Figures S1-4: Legends**

**Acknowledgments Wellcome Trust Case Control Consortium 2**

| **TABLE S1: Details of all samples used in the study** | | | | |
| --- | --- | --- | --- | --- |
| **Study** | **Cohort (Case Phenotype)** | **Array** | **# Cases** | **# Controls** |
|  |  |  |  |  |
| **Discovery** | | | | |
| WTCCC2 | Ireland (SZ) | Affymetrix 6.0 | 1,564 | 1,748 |
| WTCCC2 | UK - 58BC | Affymetrix 6.0 |  | 2,663 |
| WTCCC2 | UK - NBS | Affymetrix 6.0 |  | 2,533 |
|  |  |  |  |  |
| **Replication 1** | | | | |
| ISC | UK - Aberdeen (SZ) | Affymetrix 5.0 | 727 | 694 |
| ISC | UK - London (SZ) | Affymetrix 5.0 | 547 | 0 |
| ISC | UK - Edinburgh (SZ) | Affymetrix 6.0 | 403 | 290 |
| ISC | Portugal (SZ) | Affymetrix 5.0 | 333 | 200 |
| ISC | Sweden (SZ) | Affymetrix 5.0/6.0 | 622 | 437 |
| ISC | Bulgaria (SZ) | Affymetrix 6.0 | 479 | 646 |
|  |  |  |  |  |
| **Replication 2** | | | | |
| WTCCC1 | UK (BPD) | Affymetrix 500K | 1,697 | 10,259^a^ |
| McQuillin et al. | UK (BPD) | Affymetrix 500K | 546 | 510 |
|  |  |  |  |  |
|  |  |  |  |  |
| **Additional UK Samples** | | | | |
| CLOZUK | UK (SZ) | Illumina OmniExpress/Combo | 6,223 |  |
| WTCCC2 | UK - POBI | Affymetrix 6.0 |  | 2,507 |
|  |  |  |  |  |
| **Other European and European Ancestry Samples** | | | | |
| SGENE | Iceland (SZ) | Illumina HumanHap317/370 | 627 | 33,729 |
| SGENE | Germany (SZ) | Illumina HumanHap317/370 | 1,600 | 1,600 |
| SGENE | Holland (SZ) | Illumina HumanHap550 | 614 | 3,687 |
| SGENE | Finland (SZ) | Illumina HumanHap550 | 580 | 3,273 |
| SGENE | Denmark (SZ) | Illumina HumanHap610 | 765 | 493 |
| SGENE | Norway (SZ) | Illumina HumanHap610 | 363 | 367 |
| SGENE | UK (SZ) | Illumina HumanHap610 | 92 | 83 |
| SGENE | Italy (SZ) | Illumina HumanHap610 | 84 | 86 |
| Ripke et al. | Sweden (SZ) | Affy6.0/Illumina OmniExpress | 4,719 | 5,918 |
| Cooper et al. | US - NINDS (Coriell 500K) | HumanHap550v3_A |  | 441 |
| Cooper et al. | US - NINDS (317K +240K) | Illumina 317K+240K |  | 227 |
| Cooper et al. | US - PARC (CAP and PRINCE) | Illumina 550K |  | 936 |
| Cooper et al. | US - PARC2 (CAP2) | Illumina 550K |  | 232 |
| Cooper et al. | US - PARC2(PRINCE2) | Illumina610K Quad |  | 534 |
| Cooper et al. | US - FHCRC | Human 610v1_B |  | 1,334 |
| Cooper et al. | Italy - InChianti | Illumina HumanHap550v3_a |  | 695 |
| ARIC^b^ | US | Affymetrix 6.0 |  | 11,305 |
|  |  |  |  |  |
| Total |  |  | 22,585 | 77,168 |
| ^a^ Non-psychiatric cases from WTCCC1 | | | | |
| ^b^ For the ARIC controls the arrays were obtained from dbGAP (phs000090.v1.p1). CNV calls were performed using the Affymetrix Genotyping Console 4.1, based on a minimum of 20 probes and a minimum genomic size of 10kbp. Additionally samples with an excess number of CNV calls (> 72) were filtered as potentially unreliable samples based on an outlier detection approach for skewed data (<http://onlinelibrary.wiley.com/doi/10.1002/cem.1123/pdf>). | | | | |

| **TABLE S2: Details on WTCCC2 sample exclusions.** | | | | |
| --- | --- | --- | --- | --- |
|  | **Irish SZ Cases** | **Irish Controls** | **UK Controls (58BC)** | **UK Controls**  **(NBS)** |
| **SNP call rate <0.95** | 6 | 0 | 4 | 3 |
| **<40 sample on plate^a^** | 67 | 29 | 47 | 147 |
| **>30 large CNVs^b^** | 13 | 34 | 1 | 14 |
| **Large rare events >10Mbp^c^** | 7 | 2 | 6 | 5 |
| **SNP calling^d^** | 302 | 74 | 276 | 285 |
| **Total excluded** | 395 | 139 | 334 | 454 |
| **Final included** | 1564 | 1748 | 2663 | 2533 |
| ^a^ excluded from analysis as they were on a hybridization containing less than 40 samples. | | | | |
| ^b^ samples contained more than 30 large (>100kb) rare (<1% population frequency) CNVs. | | | | |
| ^c^ excluded because the total event length of large (>100kb), rare (<1% population frequency) CNVs was >10Mbp. | | | | |
| ^d^ samples excluded by previously described WTCCC2 SNP QC procedures. | | | | |

**TABLE S3:A: Identified carriers of known schizophrenia risk CNVs in our discovery set.B:All large (>100kb) and rare (MAF<0.01) CNVs identified in the discovery dataset.**

 See accompanying Excel file

**Table S4: Results of qPCR validation**

See accompanying Excel file with details of qPCR results at PAK7.

| **TABLE S5: All carriers of duplications at *PAK7* on chromosome 20 (hg18)** | | | | |
| --- | --- | --- | --- | --- |
| **Sample** | **Origin** | **Local ID** | **Start** | **End** |
| WTCCC2 SZ cases | Ireland | IRL_101 | 9685413 | 9831947 |
| WTCCC2 SZ cases | Ireland | IRL_201 | 9685413 | 9831947 |
| WTCCC2 SZ cases | Ireland | IRL_301 | 9685413 | 9831947 |
| WTCCC2 SZ cases | Ireland | IRL_401 | 9685413 | 9834500 |
| WTCCC2 SZ cases | Ireland | IRL_501 | 9686531 | 9829109 |
| WTCCC2 SZ cases | Ireland | IRL_601^a^ | 9685413 | 9831947 |
| ISC SZ cases | UK_Aberdeen |  | 9685413 | 9824688 |
| ISC SZ cases | UK_Aberdeen |  | 9685413 | 9829748 |
| ISC SZ cases | UK_Aberdeen |  | 9685413 | 9829748 |
| ISC SZ cases | UK_Edinburgh | SCOT_101 | 9685413 | 9834500 |
| ISC SZ cases | UK_Edinburgh |  | 9685413 | 9834500 |
| ISC SZ cases | UK_London |  | 9685413 | 9817570 |
| CLOZUK SZ cases | UK |  | 9652943 | 9747765 |
| CLOZUK SZ cases | UK |  | 9682770 | 9924721 |
| CLOZUK SZ cases | UK |  | 9682770 | 9924721 |
| CLOZUK SZ cases | UK |  | 9685413 | 9833653 |
| CLOZUK SZ cases | UK |  | 9685413 | 9833653 |
| CLOZUK SZ cases | UK |  | 9685413 | 9833653 |
| CLOZUK SZ cases | UK |  | 9685413 | 9833653 |
| WTCCC1BPD cases | UK_Cardiff |  | 9684363 | 9824688 |
| WTCCC1BPD cases | UK_London |  | 9685413 | 9861678 |
| WTCCC1BPD cases | UK_Aberdeen |  | 9685413 | 9815136 |
| McQuillin_et_al BPD cases | UK |  | 9685413 | 9840328 |
| WTCCC2 controls | UK_58BC |  | 9685413 | 9829748 |
| WTCCC2 controls | UK_NBS |  | 9608359 | 9756235 |
| WTCCC2 controls | UK_NBS |  | 9685413 | 9834500 |
| WTCCC2 controls | UK_POBI |  | 9685413 | 9829748 |
| WTCCC2 controls | UK_POBI |  | 9082549 | 9707414 |
| WTCCC1 non-psychiatric cases | UK_WTCCC1_RA |  | 9685413 | 9820828 |
| WTCCC1 non-psychiatric cases | UK_WTCCC1_HT |  | 9686531 | 9862400 |
| WTCCC1 non-psychiatric cases | UK_WTCCC1_T1D |  | 9686531 | 9815136 |
| WTCCC1 non-psychiatric cases | UK_WTCCC1_CD |  | 9686531 | 9824688 |
| WTCCC1 non-psychiatric cases | UK_WTCCC1_T2D |  | 9689876 | 9796854 |
| SGENE SZ cases | Denmark |  | 9686531 | 9833653 |
| SGENE SZ cases | Holland |  | 9688441 | 9833653 |
| SGENE controls | Denmark |  | 9682770 | 9923954 |
| SGENE controls | Holland |  | 9688441 | 9833653 |
| SGENE controls | Holland |  | 9712263 | 9833653 |
| Ripke et al SZ cases | Sweden |  | 9679193 | 9895068 |
| Ripke et al SZ cases | Sweden |  | 9682770 | 9842874 |
| Ripke et al controls | Sweden |  | 9682770 | 9919125 |
| Ripke et al controls | Sweden |  | 9689876 | 9915394 |
| Ripke et al controls | Sweden |  | 9686531 | 9831948 |
| Ripke et al controls | Sweden |  | 9682770 | 9924722 |
| Ripke et al controls | Sweden |  | 9685413 | 9829110 |
| Ripke et al controls | Sweden |  | 9686531 | 9824689 |
| Ripke et al controls | Sweden |  | 9682770 | 9917372 |
| European ancestry controls | US_Cooper_et_al |  | 9545000 | 9930000 |
| European ancestry controls | US_ARIC |  | 9684363 | 9829109 |
| European ancestry controls | US_ARIC |  | 9684363 | 9831947 |
| European ancestry controls | US_ARIC |  | 9684363 | 9831947 |
| European ancestry controls | US_ARIC |  | 9685413 | 9831947 |
| European ancestry controls | US_ARIC |  | 9685413 | 9831947 |
| European ancestry controls | US_Cooper_et_al |  | 9686531 | 9834500 |
| ^a^ IRL_601 is related to IRL_101 and thus not included in association analysis | | | | |

| **TABLE S6: Hap Block 1 and 2 core SNPs** | | | | |
| --- | --- | --- | --- | --- |
| **Hap Block** | **SNP** | **Position** | **A1** | **A2** |
| 1 | rs2423464 | 9683106 | G | C |
| 1 | rs742452 | 9684363 | C | G |
| 1 | rs742451 | 9684473 | T | C |
| 1 | rs742450 | 9684493 | T | C |
|  |  |  |  |  |
| 2 | rs6057009 | 9840327 | C | T |
| 2 | rs6516523 | 9860923 | T | C |
| 2 | rs6039636 | 9861678 | G | A |
| 2 | rs6118819 | 9867572 | G | A |

| **TABLE S7: Haplotype frequencies** | | |
| --- | --- | --- |
| **Hap Block** | **Hap** | **Freq** |
| 1 | CCTT | 0.188 |
| 1 | CCCC | 0.022 |
| 1 | GGCC | 0.301 |
| 1 | CGCC | 0.487 |
|  |  |  |
| 2 | TCAG | 0.397 |
| 2 | CTGA | 0.178 |
| 2 | CCGA | 0.089 |
| 2 | CCAA | 0.119 |
| 2 | TCAA | 0.208 |

| **TABLE S8: Phasing of haplotypes in Hap Blocks 1 and 2** | | | | |
| --- | --- | --- | --- | --- |
| **Sample** | **Hap Block 1** | | **Hap Block 2** | |
|  | **Hap 1** | **Hap 2** | **Hap 1** | **Hap 2** |
| WTCCC2 SZ cases Ireland | CCTT | CCCC | TCAA | CCAA |
| WTCCC2 SZ cases Ireland | CCTT | CGCC | TCAA | TCAA |
| WTCCC2 SZ cases Ireland | CCTT | CGCC | TCAA | TCAG |
| WTCCC2 SZ cases Ireland | CCTT | CGCC | TCAA | TCAG |
| WTCCC2 SZ cases Ireland | CCTT | CGCC | TCAA | CCGA |
| ISC SZ cases UK Aberdeen | CCTT | CGCC | TCAA | TCAA |
| ISC SZ cases UK Aberdeen | CCTT | CGCC | TCAA | CCAA |
| ISC SZ cases UK Aberdeen | CCTT | CGCC | TCAA | CTGA |
| ISC SZ cases UK London | CCTT | GGCC | TCAA | TCAA |
| ISC SZ cases UK Edinburgh | CCTT | CGCC | TCAA | CCAA |
| ISC SZ cases UK Edinburgh | CCTT | GGCC | TCAA | CTGA |
| WTCCC1 BPD cases UK Cardiff | CCTT | CGCC | TCAA | TCAG |
| WTCCC1 BPD cases UK London | CCTT | CCTT | TCAA | CTGA |
| WTCCC1 BPD cases UK Aberdeen | CCTT | CGCC | TCAA | TCAA |
| McQuillin et al BPD cases UK | CCTT | CGCC | TCAA | TCAG |
| WTCCC2 controls NBS UK | CCTT | CCTT | TCAA | TCAA |
| WTCCC2 controls 58BC UK | CCTT | CGCC | TCAA | CCGA |
| WTCCC2 controls POBI UK | CCTT | CGCC | TCAA | CTGA |
| WTCCC1 RA case UK | CCTT | GGCC | TCAA | TCAA |
| WTCCC1 CD case UK | CCTT | GGCC | TCAA | TCAG |
| WTCCC1 T2D case UK | CCTT | CCTT | TCAA | CCAA |
| WTCCC1 HT case UK | CCTT | CCTT | TCAA | CCGA |
| WTCCC1 T1D case UK | CCTT | GGCC | TCAA | CTGA |
| EA controls ARIC US | CCTT | CGCC | TCAA | TCAG |
| EA controls ARIC US | CCTT | CGCC | TCAA | TCAA |
| EA controls ARIC US | CCTT | CCTT | TCAA | CTGA |
| EA controls ARIC US | CCTT | CGCC | TCAA | CCAA |
| EA controls ARIC US | CCTT | CGCC | TCAA | TCAG |

**TABLE S9 (details on clinical features of identified PAK7 duplication carriers)**

See accompanying Excel file

**SUPPLEMENTARY FIGURE S1**

Legend: Barplot of the degree of relatedness between individuals in the analysis of ancestry within the Irish discovery sample. Number of pairs=the total number of possible pairs within the sample showing segmental sharing by IBD. The degree of relatedness for PAK7 carriers was 8, indicating no increase in genome IBD sharing in these individuals.

**SUPPLEMENTARY FIGURE S2**

Legend: aCGH analysis of PAK7 carrier. A customized array was designed using Agilent e-array with ~117,000 probes from the ISCA 4X180K group to cover the backbone of the genome and ~ 63,000 probes were chosen for high-density coverage (average spacing 150-200bp) for 18 regions, associated with common copy number variants (full details available on request). One of these high-density target regions was a region of 346.5kb on chr20 (spanning 9637413 to 9983948; Hg19). The array CGH hybridizations were carried out according to Agilent protocols found on the Agilent Technologies website
(<http://www.chem.agilent.com/Library/usermanuals/Public/G4410-90010_CGH_Enzymatic_Protocol_7.2.pdf).> A 148kb duplication of 20p12.2 was detected overlapping PAK7, with breakpoints at 9,736,902bp and 9,885,151(Hg19). These breakpoints equate to chr20:9,684,902-9,833,151(Hg18) and are very similar to those estimated from the SNP array (chr20:9,685,413-9,831,947).

**SUPPLEMENTARY FIGURE 3**

Legend: Duplication events at the chr20p12.2 locus (hg18). This is an extension of figure 1 in the main text and includes additional data from other European and European ancestry samples. The main text refers to there being 6 carriers of the duplication in the European ancestry samples from the US. This number does not include the large duplication in a sample from the Cooper et al study. It was not included in the haplotype/ancestry analysis because that analysis focused on the “common” duplication shared by most carriers.

**SUPPLEMENTARY FIGURE 4**

Legend: In order to validate the purity of our synaptosomal preparation we stained for proteins preferentially expressed in cytoplasm (MAP2) or in synapses (PSD95) in extracts from different compartments. We confirm that the MAP2 staining is preferentially restricted to cytoplasmic extracts, while the PSD95 staining is mostly present in synaptic extracts. Actin, which is present in both compartments show a comparable expression in both cytoplasm and synapses.

**Acknowledgements**

**Members of Wellcome Trust Case Control Consortium 2**

Management Committee

Peter Donnelly (Chair)^1,2^, Ines Barroso (Deputy Chair)^3^, Jenefer M Blackwell^4, 5^, Elvira Bramon^6^ , Matthew A Brown^7^ , Juan P Casas^8^ , Aiden Corvin^9^, Panos Deloukas^3^, Audrey Duncanson^10^, Janusz Jankowski^11^, Hugh S Markus^12^, Christopher G Mathew^13^, Colin NA Palmer^14^, Robert Plomin^15^, Anna Rautanen^1^, Stephen J Sawcer^16^, Richard C Trembath^13^, Ananth C Viswanathan^17^, Nicholas W Wood^18^

Data and Analysis Group

Chris C A Spencer^1^, Gavin Band^1^, Céline Bellenguez^1^, Colin Freeman^1^, Garrett Hellenthal^1^, Eleni Giannoulatou^1^, Matti Pirinen^1^, Richard Pearson^1^, Amy Strange^1^, Zhan Su^1^, Damjan Vukcevic^1^, Peter Donnelly^1,2^

DNA, Genotyping, Data QC and Informatics Group

Cordelia Langford^3^, Sarah E Hunt^3^, Sarah Edkins^3^, Rhian Gwilliam^3^, Hannah Blackburn^3^, Suzannah J Bumpstead^3^, Serge Dronov^3^, Matthew Gillman^3^, Emma Gray^3^, Naomi Hammond^3^, Alagurevathi Jayakumar^3^, Owen T McCann^3^, Jennifer Liddle^3^, Simon C Potter^3^, Radhi Ravindrarajah^3^, Michelle Ricketts^3^, Matthew Waller^3^, Paul Weston^3^, Sara Widaa^3^, Pamela Whittaker^3^, Ines Barroso^3^, Panos Deloukas^3^**.**

Publications Committee

Christopher G Mathew (Chair)^13^, Jenefer M Blackwell^4,5^, Matthew A Brown^7^, Aiden Corvin^9^, Chris C A Spencer^1^

1 Wellcome Trust Centre for Human Genetics, University of Oxford, Roosevelt Drive, Oxford OX3 7BN, UK; 2 Dept Statistics, University of Oxford, Oxford OX1 3TG, UK; 3 Wellcome Trust Sanger Institute, Wellcome Trust Genome Campus, Hinxton, Cambridge CB10 1SA, UK; 4 Telethon Institute for Child Health Research, Centre for Child Health Research, University of Western Australia, 100 Roberts Road, Subiaco, Western Australia 6008; 5 Cambridge Institute for Medical Research, University of Cambridge School of Clinical Medicine, Cambridge CB2 0XY, UK; 6 Department of Psychosis Studies, NIHR Biomedical Research Centre for Mental Health at the Institute of Psychiatry, King’s College London and The South London and Maudsley NHS Foundation Trust, Denmark Hill, London SE5 8AF, UK; 7 University of Queensland Diamantina Institute, Brisbane, Queensland, Australia; 8 Dept Epidemiology and Population Health, London School of Hygiene and Tropical Medicine, London WC1E 7HT and Dept Epidemiology and Public Health, University College London WC1E 6BT, UK; 9 Neuropsychiatric Genetics Research Group, Institute of Molecular Medicine, Trinity College Dublin, Dublin 2, Eire; 10 Molecular and Physiological Sciences, The Wellcome Trust, London NW1 2BE; 11 Department of Oncology, Old Road Campus, University of Oxford, Oxford OX3 7DQ, UK , Digestive Diseases Centre, Leicester Royal Infirmary, Leicester LE7 7HH, UK and Centre for Digestive Diseases, Queen Mary University of London, London E1 2AD, UK; 12 Clinical Neurosciences, St George's University of London, London SW17 0RE; 13 King’s College London Dept Medical and Molecular Genetics, King’s Health Partners, Guy’s Hospital, London SE1 9RT, UK; 14 Biomedical Research Centre, Ninewells Hospital and Medical School, Dundee DD1 9SY, UK; 15 King’s College London Social, Genetic and Developmental Psychiatry Centre, Institute of Psychiatry, Denmark Hill, London SE5 8AF, UK; 16 University of Cambridge Dept Clinical Neurosciences, Addenbrooke’s Hospital, Cambridge CB2 0QQ, UK; 17 NIHR Biomedical Research Centre for Ophthalmology, Moorfields Eye Hospital NHS Foundation Trust and UCL Institute of Ophthalmology, London EC1V 2PD, UK; 18 Dept Molecular Neuroscience, Institute of Neurology, Queen Square, London WC1N 3BG, UK.
