# Supplementary figures and images for: An inherited duplication at the gene p21 Protein-Activated Kinase 7 (PAK7) is a risk factor for psychosis
Source: Hum Mol Genet. 2014 Jan 28;23(12):3316–26. doi: 10.1093/hmg/ddu025 (PMC4030770; doi:10.1093/hmg/ddu025)

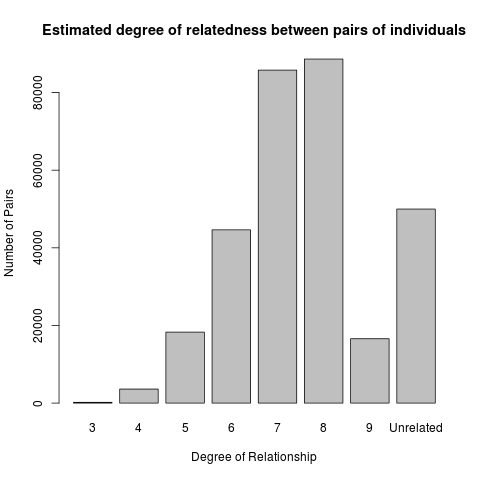

Supplement: Supplementary Data [file supp_ddu025_ddu025supp_fig1.jpg]

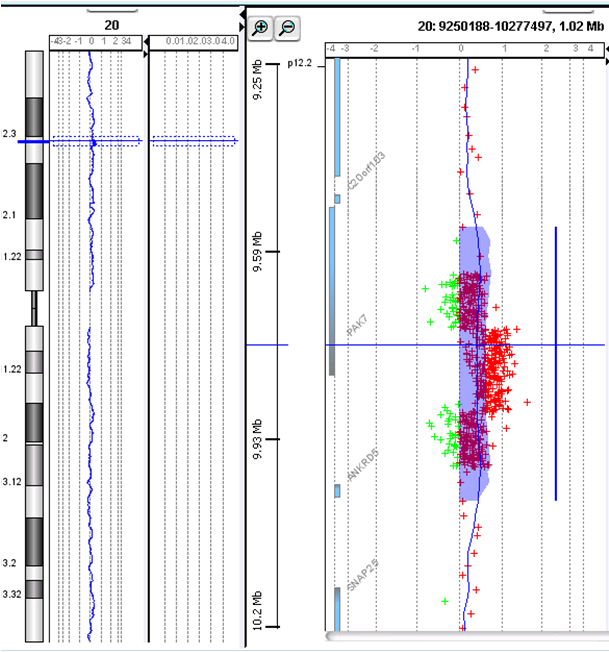

Supplement: Supplementary Data [file supp_ddu025_ddu025supp_fig2.jpg]

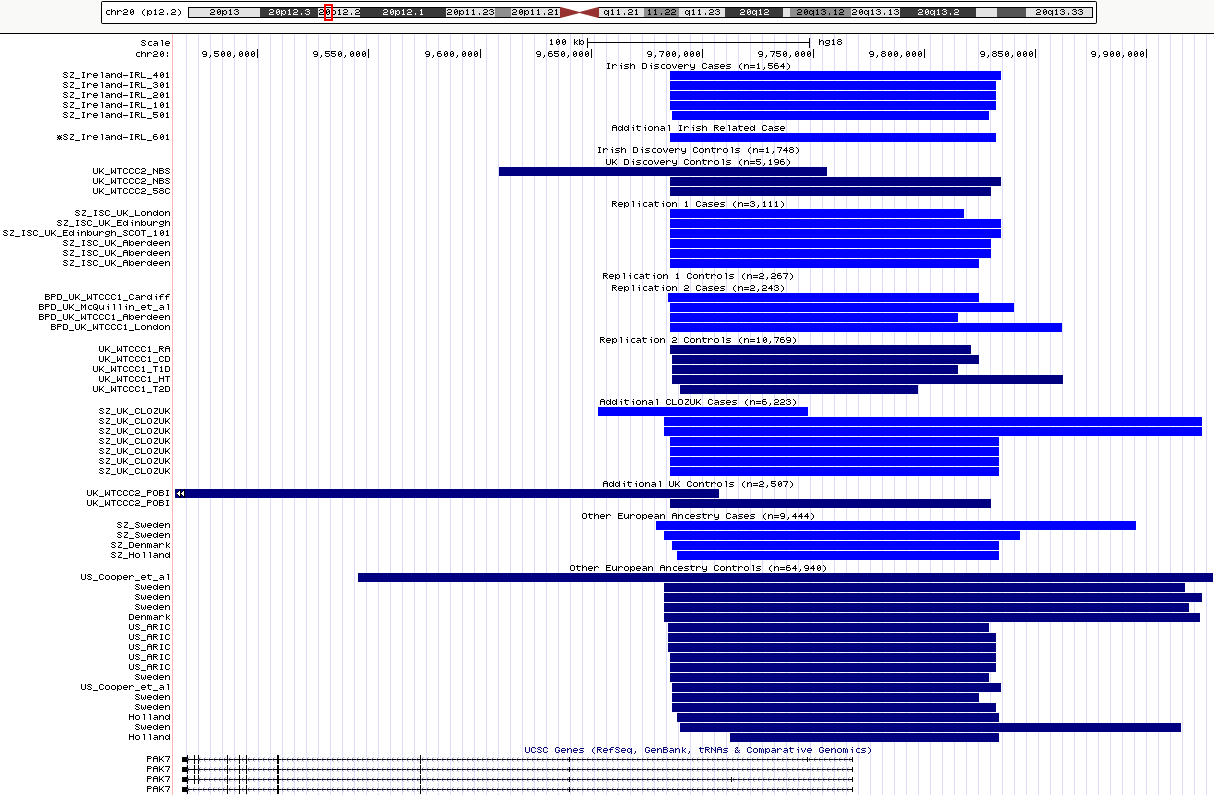

Supplement: Supplementary Data [file supp_ddu025_ddu025supp_fig3.jpg]

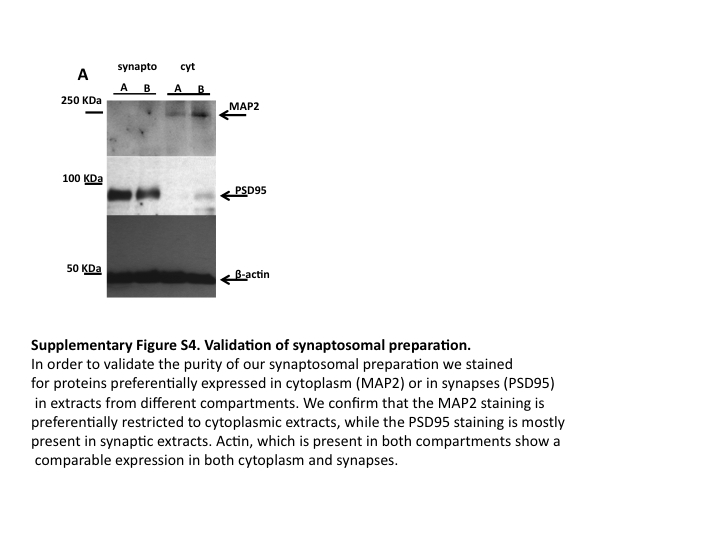

Supplement: Supplementary Data [file supp_ddu025_ddu025supp_fig4.jpg]
